# Theoretical Study of Quaternary nBp InGaAsSb SWIR Detectors for Room Temperature Condition

**DOI:** 10.3390/ma17225482

**Published:** 2024-11-10

**Authors:** Tetiana Manyk, Jarosław Rutkowski, Małgorzata Kopytko, Krzysztof Kłos, Piotr Martyniuk

**Affiliations:** 1 Institute of Applied Physics, Military University of Technology, 2 Kaliskiego St., 00-908 Warsaw, Poland; tetjana.manyk@wat.edu.pl (T.M.); jaroslaw.rutkowski@wat.edu.pl (J.R.); malgorzata.kopytko@wat.edu.pl (M.K.); 2 Photin sp. Z O.O, 15 Lutosławskiego Street, 05-080 Klaudyn, Poland; kk@photin.eu

**Keywords:** SWIR, IR detectors, barrier detectors, InGaAsSb, AlGaAsSb

## Abstract

This paper presents a theoretical analysis of an nBp infrared barrier detector’s performance intended to operate at a room temperature (300 K) based on A^III^B^V^ materials—In_1-*x*_Ga*_x_*As_y_Sb_1−*y*_ quaternary compound—lattice-matched to the GaSb substrate with a p-n heterojunction ternary Al_1−*x*_Ga*_x_*Sb barrier. Numerical simulations were performed using a commercial Crosslight Software—package APSYS. The band structure of the nBp detector and the electric field distribution for the p-n heterojunction with and without a potential barrier were determined. The influence of the barrier-doping level on the detector parameters was analyzed. It was shown that Shockley-Read-Hall (SRH) recombination plays a decisive role in carrier transport for lifetimes shorter than 100 ns. The influence of the absorber/barrier thickness on the detector’s dark current density and photocurrent was investigated. It was shown that valence band offset does not influence the device’s performance. The quantum efficiency reaches its maximum value for an absorber’s thickness of ~3 μm. The performed simulations confirmed the possibility of the detector’s fabrication exhibiting high performance at room temperature based on quaternary compounds of A^III^B^V^ materials for the short wavelength infrared range.

## 1. Introduction

A^III^B^V^ compound materials are suitable for optoelectronic devices fabrication in the near- (NIR), mid- (MWIR), and long-infrared wavelength (LWIR) ranges [1,2,3,4,5,6,7,8,9,10,11,12]. The short-wavelength infrared region from 1 to 3 µm (SWIR) detectors are a valuable addition to imaging technology, providing several unique features not available in the standard night vision devices. SWIR detectors, compared with visible (VIS) devices, can detect clear images even in a fog or smoke environment. The high-performance detectors covering VIS and SWIR regions are also useful in monitoring and remote sensing. Moreover, the SWIR detectors operate by detecting the reflections from objects [4]. The SWIR image sensors provide low-light-level imaging [8]. Devices that allow for reaching high operating temperature (HOT) with low dark current and high quantum efficiency (QE) are desirable for many applications [7,13,14]. HOT SWIR detectors can significantly reduce the cost of cryogenic cooling, which reduces the detector system’s size, weight, power, and total cost (SWaP condition). A shortcoming of ternary InGaAs arises due to the shorter cut-off wavelength of 1.8 µm. On the other hand, InGaSb ternary material indicated good performance for 2 µm. The wavelength of 2–3 µm is a performance gap for infrared (IR) detectors. The existing approach for a 2–3 µm SWIR detector is the InGaAs active layer grown on InP substrates, using graded AlInAs buffer layers. It was reported that the devices based on the InGaAs grown on the InP substrate suffer from threading dislocation (caused by the lattice mismatch), increasing the dark current. IR detectors based on quaternary materials show strong potential for developing IR sensors with improved performance [4,6,10,11,12]. The availability of binary substrates such as GaSb allows for the growth of multilayer homo- and hetero-structures, where lattice-matched quaternary layers could be tailored to detect wavelengths in the range from NIR to MWIR. Quaternary systems with the flexibility of varying the cut-off wavelength by changing the molar composition of the In and As have great importance for a variety of civil and military applications, including atmospheric remote sensing, and industrial areas, such as gas detection [1,5,11]. InGaAsSb detectors grown on a GaSb substrate provide superior detectivity (D^*^) as InGaAs detectors [1]. The quaternary systems allow for a wider bandgap than binary InAs and ternary InAsSb. In addition, the AlGaAsSb layer could be used as a barrier layer to further reduce the dark current [10,14,15,16]. The quaternary structures based on the 6.1-Å family (including InAs, InSb, GaSb, AlSb, and their compounds) have the capability of bandgap and band offset tunability, allowing for designing and fabricating the barrier devices.

Devices based on InGaAsSb have shown their significance in improving the operating temperature in both SWIR and MWIR regions. The IR detectors based on reverse-biased homojunction and heterojunction p-n diodes are extensively used since the built-in electrostatic barrier blocks the flow of majority carriers; however, simple p-n junction devices suffer from SRH dark current because of the activation of mid-gap centers in the depletion region. A large electric field in the depletion region causes unoccupied mid-gap centers to become partially filled due to band bending that ultimately results in high dark currents. To suppress the SRH or generation and recombination (G-R) dark current components, as well as any leakage of majority carriers from the absorber into the collector, the barrier-based detector architecture with InGaAsSb absorbing and collector layers was used. An AlGaAsSb-based compound is implemented as an electron barrier to suppress the majority-carrier current and to control the electric field in the absorber. The barrier, adjacent to the absorber, needs to be optimized to minimize the electric field, hence, lowering the G-R dark current and suppressing the majority carrier current while not impeding the minority carrier transport [10,16,17].

This paper provides a theoretical analysis of the room temperature *I*-*V* characteristics for nBp aluminum (Al) barrier detectors with an active layer based on quaternary In_0.14_Ga_0.86_As_0.10_Sb_0.90_ bulk materials and a ternary Al_0.20_Ga_0.80_Sb barrier layer. The influence of the barrier layer and the active layer doping/thickness on the *I*-*V* characteristics is presented. So far, the published literature has presented research on the detectors based on the *x_In_* = 0.28 indium (In) composition [14]. Reducing the In fraction to 0.14 shifts the sensitivity edge to 2.2 μm and reduces dark currents. A quaternary material with this *x_In_ =* 0.14 composition is promising for use in SWIR detection.

## 2. nBp Photodiode Structure and Theoretical Modeling Approach

The modeling was performed using a commercial Crosslight Software-simulation package APSYS (SimuApsys2019). APSYS offers a very flexible simulation environment for modern semiconductor devices [18]. It consolidates all the device structural information as well as the material and simulation parameters. Material data used in the modeling are given in [2,3,11,15,16,19,20]. The In_1−*x*_Ga*_x_*As_y_Sb_1−*y*_ composition-dependent (*x*, *y*) bandgap energy can be reached with the approximation through the corresponding bandgap energy of the binary compounds [15,20]: InAs, InSb, GaAs, and GaSb can be given by the following relation [19,21]:

(1) $$E_{g}^{InGaAsSb}\left(x,y\right)=xyE_{g}^{InAs}+x\left(1-y\right)E_{g}^{InSb}+\left(1-x\right)yE_{g}^{GaAs}+\left(1-x\right)\left(1-y\right)E_{g}^{GaSb}$$

To include the nonlinearity in the bandgap, the bowing contribution should be added to the corresponding linear formula in Equation (2):

(2) $${bowE}_{g}^{InGaAsSb}\left(x,y\right)=-x\left(1-x\right)\left[yC_{GaInAs}+\left(1-y\right)C_{GaInSb}\right]-y\left(1-y\right)\left[xC_{GaAsSb}+\left(1-x\right)C_{InAsSb}\right]$$

where *C*_GaInAs_, *C*_GaInSb_, *C*_GaAsSb_, and *C*_InAsSb_ are the bowing parameters [20].

The electron affinity for the absorber and barrier layer was assumed at the levels 3.13 eV and 3.32 eV, respectively. The valence band offset (VBO) between the barrier and InGaAsSb-active layer was assumed at the level –0.19 eV.

The effective masses for binary compounds (InAs, InSb, GaAs, and GaSb) are summarized in Table 1. Bowing contribution to the effective mass should be added to include the nonlinearity resulting from the nonlinear dependent bandgaps of ternary alloys [20]. The carrier-effective masses for In_1−x_Ga*_x_*As*_y_*Sb_1−*y*_ lattice-matched to GaSb can be calculated as follows:

(3) $$m_{e}/m_{0}=0.0294{\left(1-x\right)}^{3}-0.0378{\left(1-x\right)}^{2}-0.0122\left(1-x\right)+0.039,$$

(4) $$m_{hh}/m_{0}=-0.1376{\left(1-x\right)}^{3}+0.0259{\left(1-x\right)}^{2}+0.1235\left(1-x\right)+0.4001,$$

(5) $$m_{lh}/m_{0}=0.0075{\left(1-x\right)}^{3}-0.0143{\left(1-x\right)}^{2}-0.0182\left(1-x\right)+0.05.$$

Electron and hole mobilities, *µ_e_* and *µ_h_*, are influenced by several mechanisms, such as scattering by interaction with thermal lattice vibrations, charged or neutral impurities (dopants), lattice defects, and surfaces. These important effects must be simulated without generating an endless number of parameters. Thus, carrier mobility modeling compiles the current transport properties and their dependence on relevant parameters, such as carrier temperatures, lattice temperature, dopant concentrations, material composition and driving electric fields. For electrons, the transferred electron model (*n.gaas* model, used in many A^III^B^V^ semiconductors) [18], exhibiting negative differential resistance due to the transition of carriers into band valleys with lower mobility, was implemented. The mobility model for this case is given by the following relation:

(6) $$\mu_{e}=\frac{\mu_{0e}+\left(v_{e}^{sat}/F_{0e}\right){\left(F/F_{0e}\right)}^{3}}{1+{\left(F/F_{0e}\right)}^{4}}.$$

For holes, we adopted the Canali model (Beta model) [22], which is commonly used in the literature and was implemented in the following form:

(7) $$\mu_{h}=\frac{\mu_{0h}}{{\left[1+{\left(\mu_{0h}F/v_{h}^{sat}\right)}^{\beta_{h}}\right]}^{1/\beta_{h}}},$$

where *µ*_0*e*_ and *µ*_0*h*_ are constant mobilities for electrons and holes, $v_{e}^{sat}$ and $v_{h}^{sat}$ are the bulk saturation velocities for electrons and holes [22], and *F*_0*e*_ is a threshold field beyond which the electron velocity saturates.

The absorption coefficient was determined based on the following model [15]:

(8) $$\alpha \left(h\nu \right)=\alpha_{g}{\left[\frac{ln\left(1+e^{\left(h\nu -E_{g}\right)/pE_{u}}\right)}{ln2}\right]}^{p},$$

(9) $$p=p_{g}+\frac{h\nu -E_{g}}{E_{m}},$$

where *E_g_* is the bandgap energy, *E_u_* = 0.02 eV stands for the characteristic Urbach energy based on the slope of the absorption tail below the bandgap, *α_g_* = 5.5 × 10^5^ m^−1^ is the magnitude of the absorption coefficient at the bandgap energy, and *p_g_* = 0.15 is a constant term describing the power law at the bandgap and a photon-energy-dependent term *hν* that describes the variation in the power law above the bandgap with characteristic energy *E_m_* = 2 eV.

**Figure 1:**
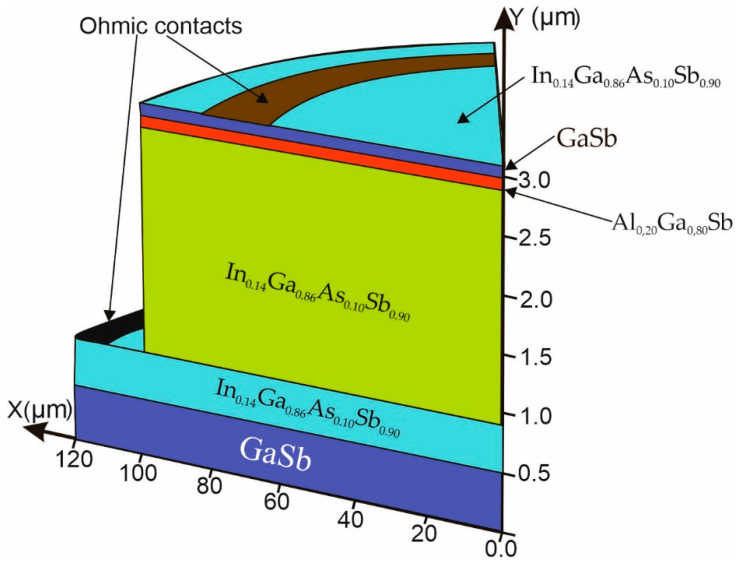
The structure of the analyzed nBp barrier detector.

The paper analyzes the performance of nBp barrier IR detectors based on a quaternary compound made of A^III^B^V^ materials, In_1−*x*_Ga*_x_*As*_y_*Sb_1−*y*_ [16], lattice-matched to a GaSb substrate. The assumed In (0.14) and As (0.1) compositions provided a target SWIR-operating wavelength of 2.2 μm. The barrier was the ternary compound Al_1–*x*_Ga*_x_*Sb, with thickness below a critical value, which would cause layer relaxation. A detector for the SWIR range operating at a temperature of 300 K with the structure given below was selected for analysis. The structure included an N^+^ 500 nm thick buffer GaSb layer, doped at the level of *n* = 2 × 10^18^ cm^−3^, a lower contact with a thickness of 400 nm made of the quaternary In_0.14_Ga_0.86_As_0.10_Sb_0.90_ material doped with n-type tellurium *n* = 2 × 10^18^ cm^−3^, an absorber layer (AL) made of the same material and doped to the level of *n* = 2 × 10^17^ cm^−3^ with a thickness of 2000 nm, a barrier layer (BL) made of a ternary Al_0.20_Ga_0.80_Sb material with a thickness of 100 nm and the upper contact layer build of 100 nm GaSb, and 5 nm In_0.14_Ga_0.86_As_0.10_Sb_0.90_ p-type doped at the level of 2 × 10^18^ cm^−3^. The schematic 3D structure of the analyzed detector is shown in Figure 1, and the main parameters are presented in Table 2.

**Figure 2:**
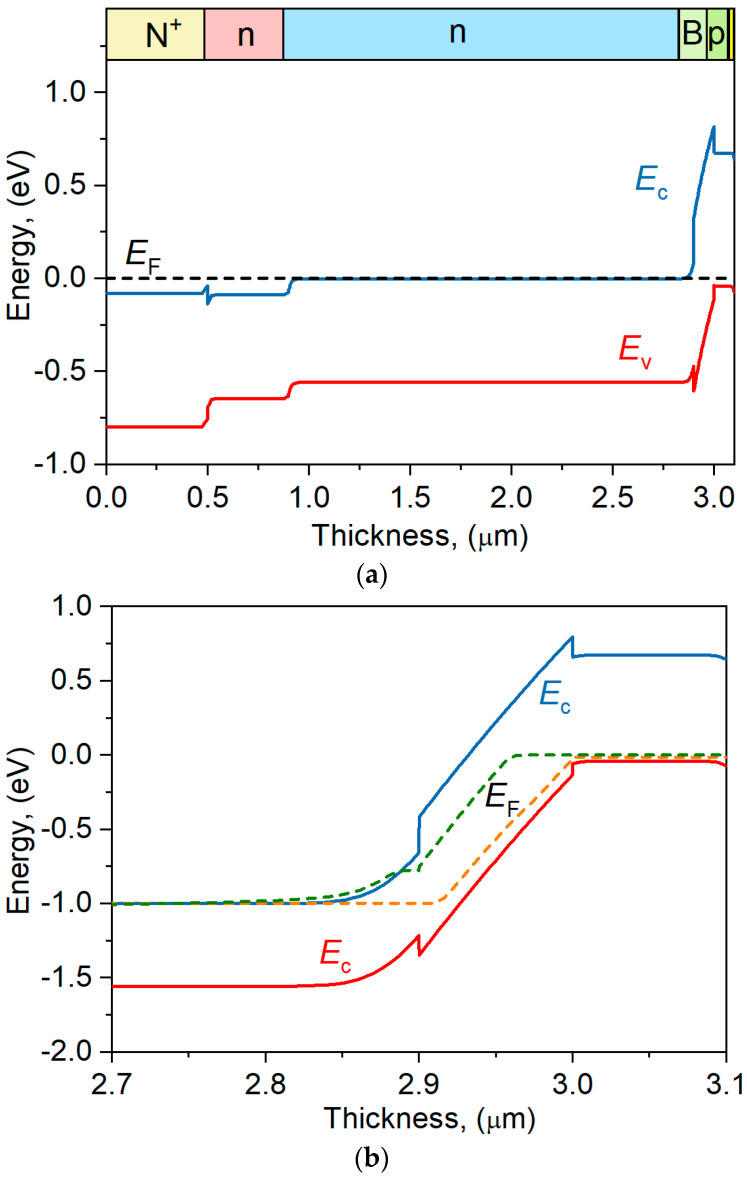
The band structure of the nBp barrier detector at 300 K under *U* = 0 V (**a**) and *U* = –1 V (**b**). Region near the barrier.

The energy band profile of the nBp detector along with a description of the individual layers is shown in Figure 2. In this structure, an electron barrier is created, which only allows for the transport of minority carriers (holes) from the absorption region to the upper contact while blocking the electrons transport. However, in the analyzed case, there is also a small potential barrier in the valence band, which may block the flow of minority holes and thus reduce the photocurrent. This barrier does not vanish at reverse bias *U* = –1 V. The voltage applied in the reverse direction mostly drops in the barrier, slightly penetrating the absorber region.

The observed potential barrier may result from the absorber and barrier parameters assumed in the simulations [10,13,14,16,23,24,25,26]. Slight changes in the composition of those materials do not cause a significant change in the height of that potential barrier in the valence band.

## 3. Results and Discussion

**Figure 3:**
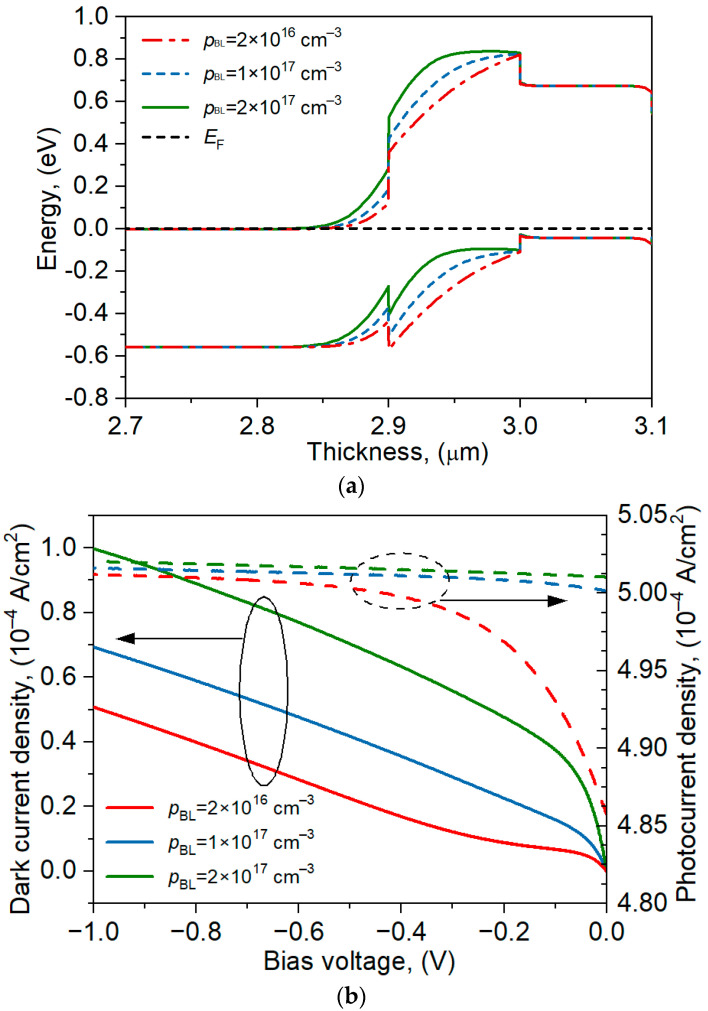
The energy bands near the barrier (**a**) and the dark current and photocurrent under reverse voltage (**b**) for three levels of the barrier’s doping, *p_BL_* = 2 × 10^16^ cm^−3^, 1 × 10^17^ cm^−3^ and 2 × 10^17^ cm^−3^.

The influence of the barrier-doping level on the shape of the bands and the photocurrent was analyzed. The detector was illuminated with radiation of wavelength *λ* = 2 μm and power *P* = 10 W. Figure 3a shows the band structure near the barrier for three p-type doping levels: *p_BL_* = 2 × 10^16^ cm^−3^, 1 × 10^17^ cm^−3^, and 2 × 10^17^ cm^−3^. The increase in the barrier doping changes its shape but does not affect the size of the potential barrier in the valence band. The higher doping of the barrier causes the electric field to penetrate the absorber layer more strongly [13,14]. The dark-current density under the reverse voltage of *U* = –1 V increases twice as the barrier doping increases by an order of magnitude (Figure 3b), while the photocurrent practically does not change (increase only by 0.16%). It follows that the doping of the barrier should remain as low as possible. The selected *p_BL_* = 2 × 10^16^ cm^−3^ as the lower limit resulted from the fact that it is difficult to grow layers with a lower doping. Higher concentrations can be obtained by introducing Be as an acceptor dopant.

**Figure 4:**
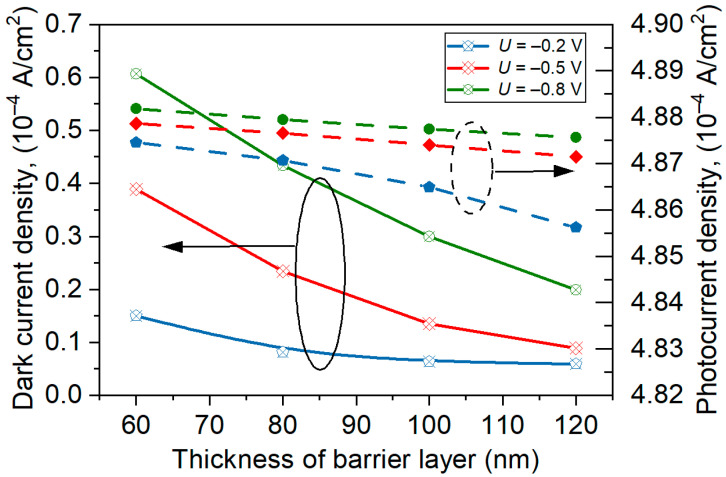
Dark current and photocurrent density versus BL thickness for reverse bias –0.2 V, –0.5 V, and –0.8 V.

An analysis of the influence of BL thickness on the detector current at 300 K was performed. Figure 4 shows the current versus BL thickness for three reverse voltages: –0.2 V, –0.5 V, and –0.8 V. The barrier thickness increases, leading to the dark current lowering, and those changes are stronger for higher voltages. The photocurrent practically does not depend on the BL thickness. The contact layers’ doping level also did not significantly affect the detector’s dark current and photocurrent.

**Figure 5:**
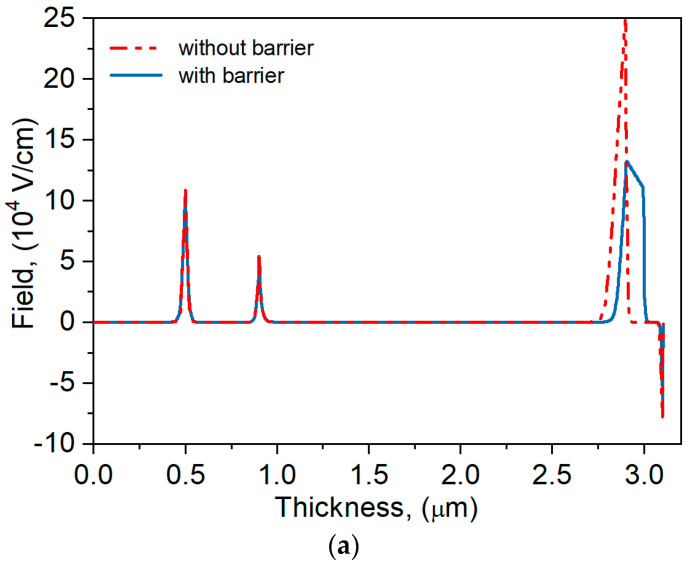

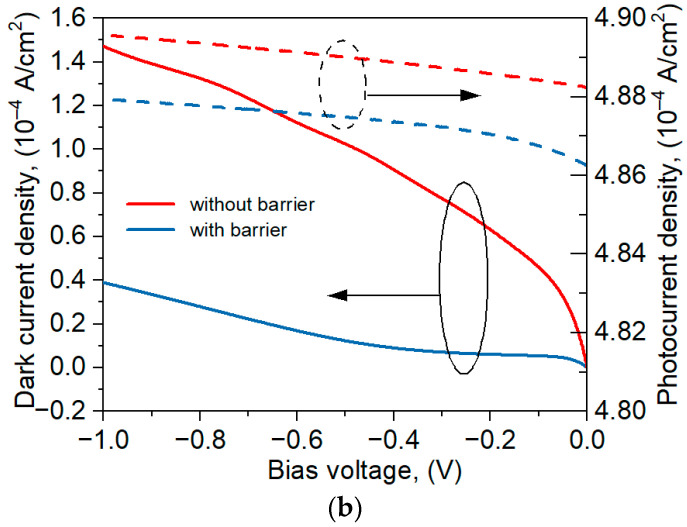
Electric field distribution (**a**) and dark current and photocurrent density under reverse bias (**b**) for the nBp detector with and without barrier layer.

To assess the BL contribution in the analyzed structure, Figure 5 compares the electric field distribution and the *I-V* characteristics shape for the detector with and without the BL. The electric field (see Figure 5a), similarly to [13], drops in the barrier region and penetrates less into the absorber layer than when there is no BL. Adding a barrier BL reduces the dark current several times but does not significantly affect the photocurrent (Figure 5b). The use of a BL, with the potential barrier occurrence in the valence band, significantly improves the detection parameters of the analyzed nBp detector.

**Figure 6:**
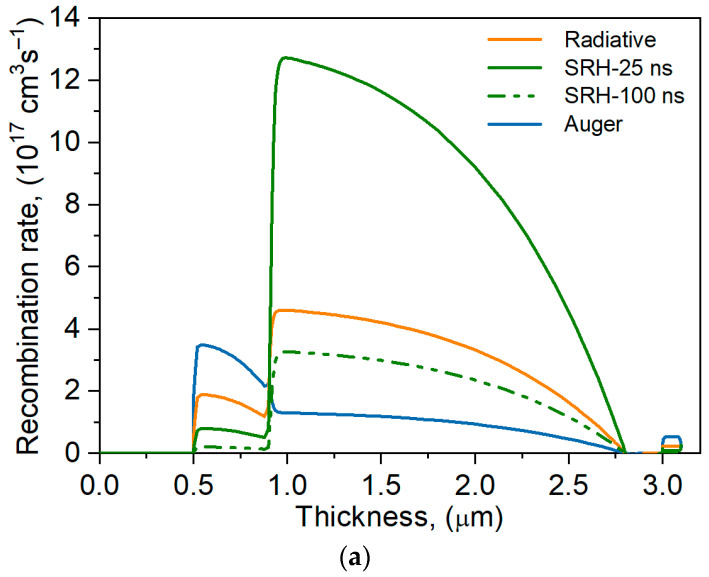

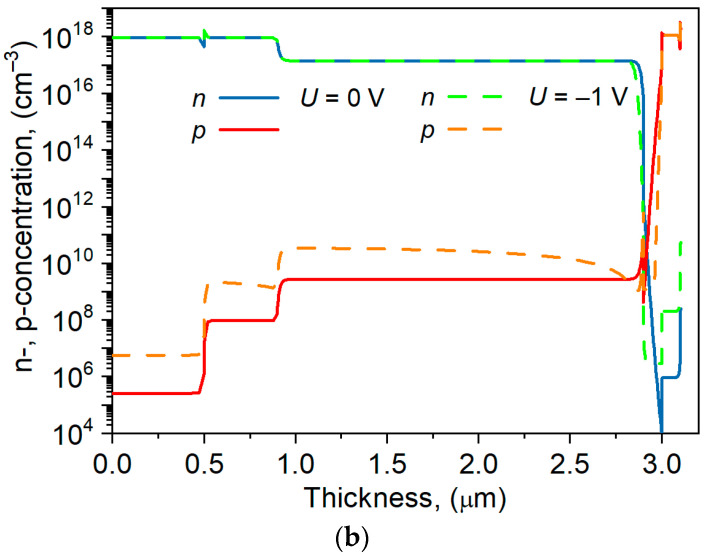
Distribution of carrier recombination rates (**a**) and electron and hole concentrations under *U* = 0 V and –1 V (**b**) for the nBp detector at room temperature.

The parameters of the analyzed detector are significantly influenced by G-R processes occurring in the device’s active layers. The level of Auger and radiation G-R was assumed for simulations based on literature data to include the following [10,17]: Auger coefficient C_n,p_ = 10^−28^ cm^6^/s and radiative coefficient *B* = 10^−10^ cm^3^/s, respectively. The SRH recombination lifetime was varied within a wide range: 1 ns÷1 µs. Figure 6a shows changes in the carrier recombination rate in the analyzed structure for two SRH lifetimes: 25 ns and 100 ns, respectively. For short lifetimes, SRH plays a decisive role in the absorber layer, while for SRH times longer than 100 ns, radiative recombination begins dominating. In the lower contact layer, Auger recombination was found to reach the highest values. The decrease in the recombination rate near the barrier results from the extraction of minority carriers (holes) from the absorber region by the p-n heterojunction. This is confirmed by the graph of the electron and hole concentration distribution at zero voltage and for the reverse bias *U* = –1 V, shown in Figure 6b. Near the p-n heterojunction, the concentration of excess holes decreases by more than an order of magnitude, and the concentration of electron majority carriers does not change.

**Figure 7:**
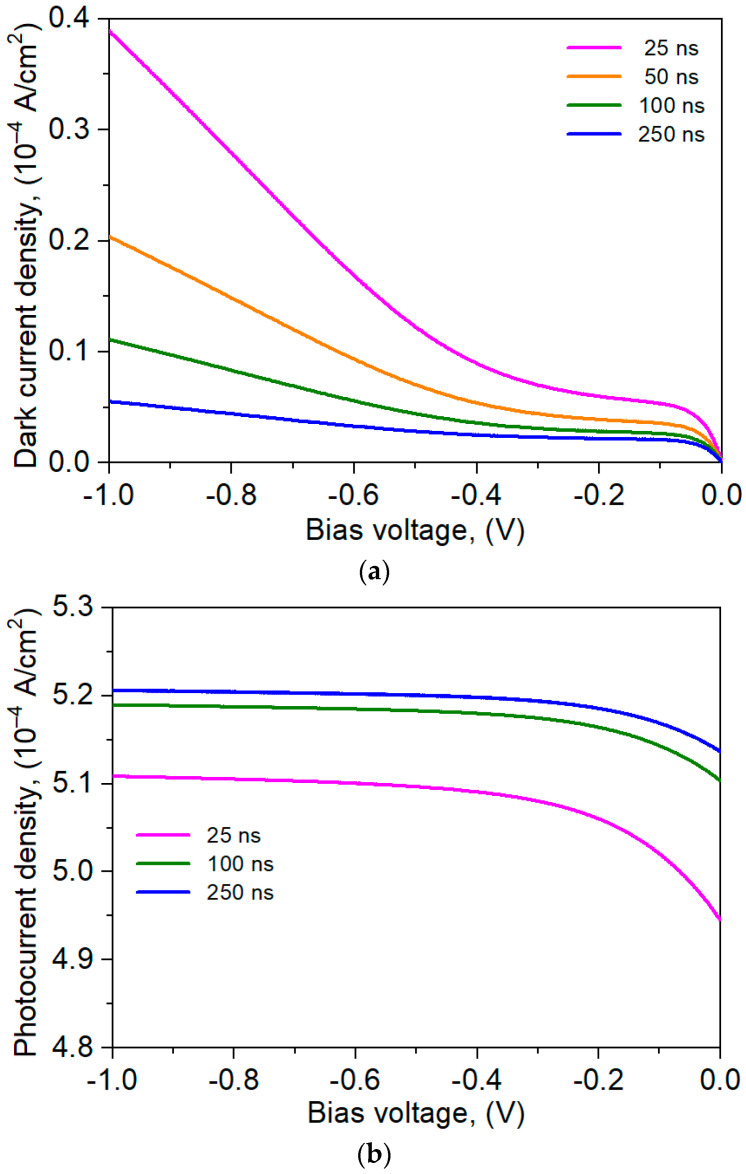
Influence of SRH carrier lifetime on the dark current (**a**) and photocurrent densities (**b**) for nBp barrier detector under reverse bias.

The carrier lifetime determined by SRH recombination significantly affects the shape of the *I-V* characteristics. Figure 7 shows the theoretical simulation of the current density dynamics versus reverse bias for selected SRH lifetimes at 300 K. The increase in the SRH lifetime causes a decrease in the dark current, especially under higher voltages (Figure 7a), while the photocurrent density increases (Figure 7b), but that increase is small, reaching ~2% when the lifetime changes by an order of magnitude. The Figure shows that for shorter SRH time, the larger dark current was found, and the dark current decreased versus the SRH carrier lifetime. The SRH carrier lifetime rise from 25 ns to 50 ns causes the dark current to change to ~0.2 × 10^−4^ A/cm^2^, while the SRH time variation from 50 ns to 100 ns leads the dark current to change to ~0.1 × 10^−4^ A/cm^2^ for 1 V reverse voltage. The SRH time increase to 250 ns causes a smaller change in dark current (0.05 × 10^−4^ A/cm^2^). Analyzing the photocurrent versus voltage for selected SRH lifetimes (25, 100, 250 ns), an inverse behavior was observed. Figure 6b shows that varying the SRH time within the range 25–100 ns causes a change in the photocurrent ~0.8 × 10^−5^ A/cm^2^, while the variation in the SRH time from 100 ns to 250 ns causes a change in the photocurrent of the order ~0.1 × 10^−5^ A/cm^2^. That allows us to conclude that the SRH time above 250 ns in the analyzed device does not improve the detector performance due to the very small change in the photocurrent compared to the SRH carrier lifetime of 100 ns. This is because for the SRH time greater than 100 ns, the carrier recombination rate is determined by other recombination mechanisms.

**Figure 8:**
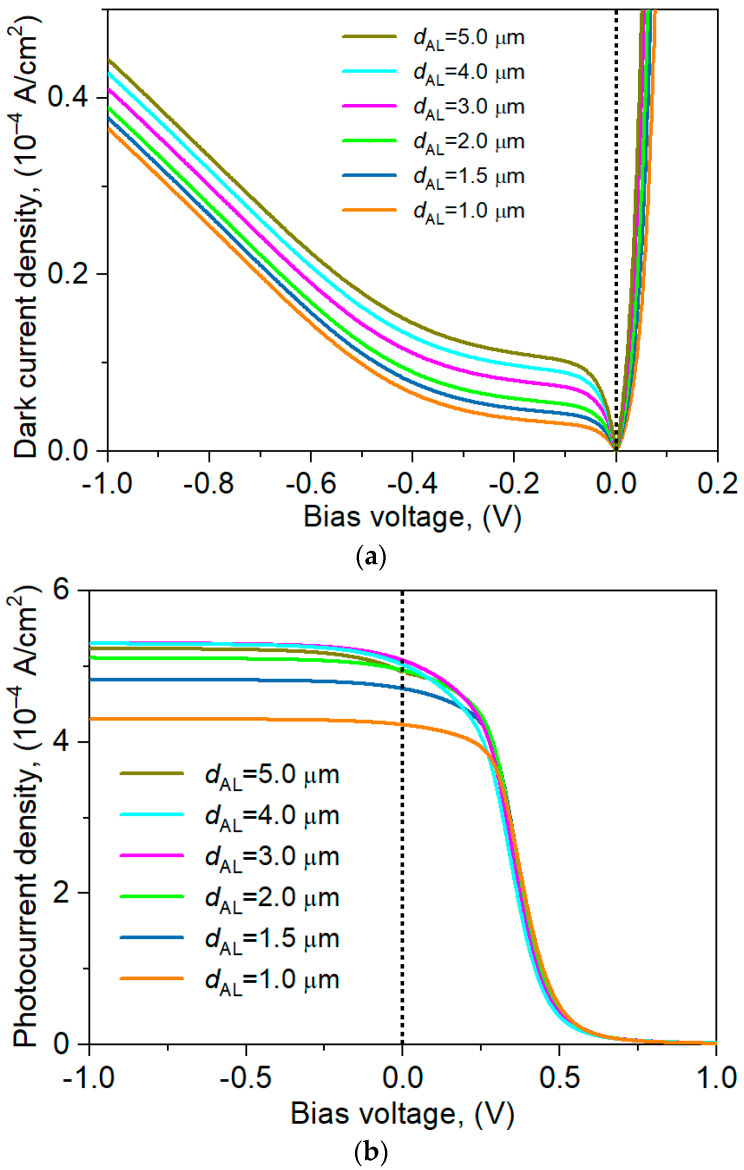
Influence of absorber thickness on the dark current (**a**) and photocurrent densities (**b**) for the nBp barrier detector.

The influence of the absorber layer thickness on the dark current and photocurrent densities for the nBp detector was analyzed, and the results are presented in Figure 8. Analyzing Figure 8a, it can be concluded that a linear change in the active layer thickness causes a linear change in the dark current. At the same time, as shown in Figure 8b, changing the active layer thickness from 1 µm to 2 µm causes a change in the photocurrent by ~1 × 10^−4^ A/cm^2^, while varying the active layer within the range 2–3 µm gives an almost negligible change in the photocurrent (0.1 × 10^−4^ A/cm^2^). As the absorber thickness increases, both the dark current and the photocurrent densities also increase, but photocurrent only up to *d*_AL_ ~3 µm. For thicknesses AL = 4 and 5 μm, the photocurrent decreases because not all carriers generated in the absorber layer reach the junction. The increase in dark current density results from the increase in the thermal generation region. From this analysis, it can be concluded that the active layer thickness ~3 µm is optimal for the analyzed device structure.

**Figure 9:**
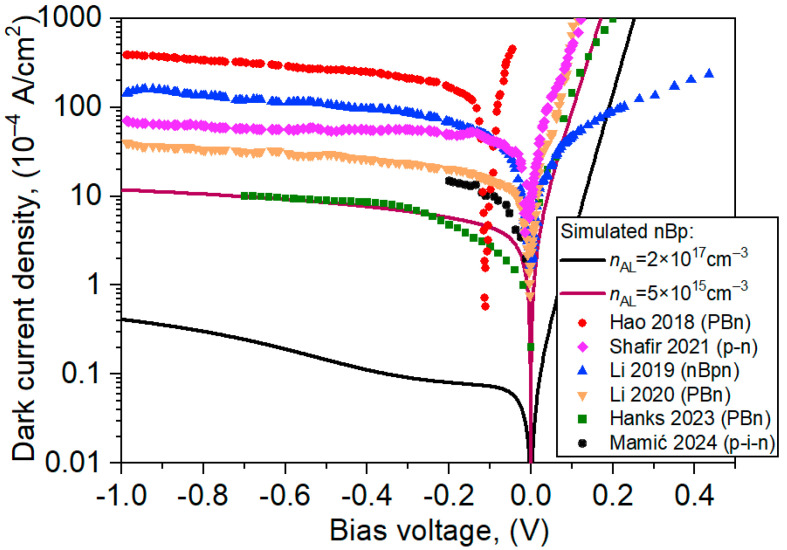
Comparison of theoretical *I-V* characteristics for analyzed nBp barrier detectors with literature data [4,12,13,14,23,27].

Figure 9 compares simulated theoretical *I-V* characteristics for analyzed nBp barrier detectors with literature data according to the References [4,12,13,14,23,27]. The reported literature data do not correspond to the analyzed structure, i.e., with the same stoichiometric composition of the absorber and the level and type of its doping, the presented nBp barrier detector reaches a significantly lower dark current. The proposed structure is characterized by a dark current lower by several orders of magnitude (curve *n*_AL_ = 2 × 10^17^ cm^−3^). If the absorber doping is reduced (curve *n*_AL_ = 5 × 10^15^ cm^−3^), the theoretical and experimental data were found to be comparable. In particular, the results (Hanks 2023—PBn) show that *x_In_* = 0.14 composition almost reduces the dark current to the theoretical level [23]. The authors show, however, that the experimentally obtained dark currents are overestimated by the significant contribution of leakage currents [27]. The decrease in carrier concentration in AL from 2 × 10^17^ cm^−3^ to 5 × 10^15^ cm^−3^ leads to the SRH recombination rate increase by an order of magnitude, contributing to the dark current.

**Figure 10:**
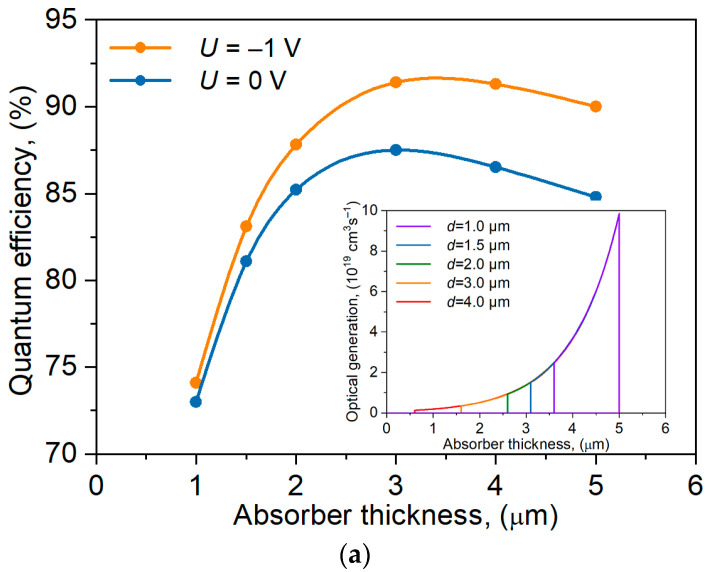

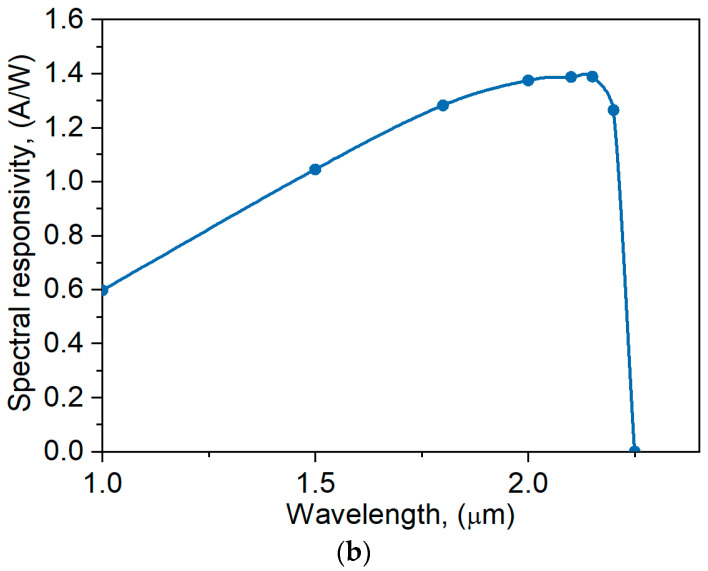
Influence of the absorber thickness on the QE and optical carrier generation (**a**); the spectral responsivity at *U* = 0 V (**b**) for the nBp detector with *d*_AL_ = 2 μm.

Based on the calculated photocurrent, the investigated detector QE was estimated in the case of illumination of the entire structure from the upper contact side (*λ* = 2 μm and *P* = 10 W) without considering the reflection of radiation from the surface and the substrate. The dependence of the QE on the absorber thickness is shown in Figure 10a under 0 and −1 V voltages. The maximum QE is for the absorber’s thickness ~3 µm. The QE slightly increases for the detector under the reverse bias [7,13,14,16]. Optical carrier generation for the selected absorber thickness is shown in Figure 10a inset. An increase in the absorber thickness (the number of optically generated carriers increases) moves the region of highest generation from the junction, leading to the QE decrease for λ > 3 μm.

Figure 10b shows the shape of the spectral characteristics of the tested nBp detector with absorber thickness *d*_AL_ = 2 μm at room temperature. The maximum current responsivity of the detector assumes ~1.4 A/W and is reached for the wavelength of 2.2 μm. The assumed dependence of the absorption coefficient on the energy of the incident radiation determines the shape of this characteristic. The specific detectivity, D*, was calculated based on Equation (10), where *R_i_* is the detector responsivity, *J* is the dark current density, *RA* is the differential resistance area product, *k* is the Boltzmann constant, and *T* is the operating temperature:

(10) $$D*=R_{i}{\left(2qJ+4kT/RA\right)}^{-1/2}$$

**Figure 11:**
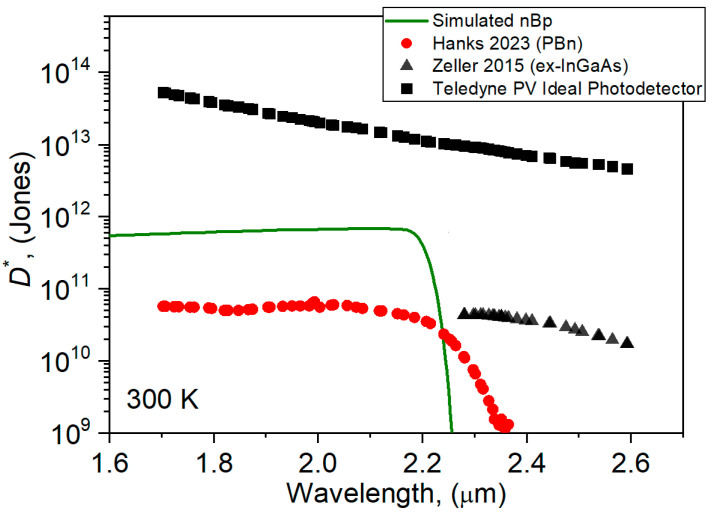
Comparison of the calculated D^*^ for nBp barrier detector with literature data [23,28,29].

The nBp detector exhibits ~6.7 × 10^11^ cmHz^1/2^W^−1^ peak detectivity at a wavelength of 2.2 μm, −0.1 V bias, and at 300 K. The determined value of D* is higher in comparison to the reported data according to Reference [23]. In practice, the experimental D* is an order of magnitude lower than our theoretical value due to low QEs and the higher noise. The presented experimental data are related to the detector with a different nBp structure and different levels of AL doping. A comparison of the calculated D* with the literature data is presented in Figure 11.

## 4. Conclusions

This paper presents an analysis of the detection parameters of nBp IR barrier detectors based on a quaternary A^III^B^V^ material, In_1−*x*_Ga*_x_*As_y_Sb_1−*y*_, which was lattice-matched to the GaSb substrate and operating at a temperature of 300 K. The strained ternary Al_0.2_Ga_0.8_Sb barrier layer was implemented, with a 100 nm thickness which could be grown without relaxation. A significant impact of the barrier on the detector parameters was demonstrated despite the presence of a slight discontinuity in the valence band. The doping level of the barrier affects the value of the dark current and should be as low as possible. SRH recombination plays a decisive role in carrier transport for lifetimes shorter than 100 ns. The optimal thickness of the absorber layer in terms of quantum efficiency was estimated at the level of ~3 μm. The performed simulations confirmed the possibility of developing detectors with high detection parameters at room temperatures made of quaternary A^III^B^V^ materials.

## Figures and Tables

**Table 1:** Effective electron and hole masses for the InAs, InSb, GaAs, and GaSb at *T* = 300 K.

Materials	*m*_e_/*m*_0_	*m*_h_/*m*_0_
InAs	0.22	0.41
InSb	0.014	0.43
GaAs	0.0635	0.51
GaSb	0.039	0.40

**Table 2:** The main parameters of the nBp barrier detector at *T* = 300 K.

Layer	Material	Thickness (nm)	Doping Concentration (cm^−3^) /type	Energy Gap (eV)	Affinity (eV)
6	In_0.14_Ga_0.86_As_0.10_Sb_0.90_	5	2 × 10^18^/p	0.56	3.13
5	GaSb	100	2 × 10^18^/p	0.72	2.55
4	Al_0.20_Ga_0.80_Sb	100	2 × 10^16^/p	0.93	3.32
3	In_0.14_Ga_0.86_As_0.10_Sb_0.90_	2000	2 × 10^17^/n	0.56	3.13
2	In_0.14_Ga_0.86_As_0.10_Sb_0.90_	400	2 × 10^18^/n	0.56	3.13
1	GaSb	500	2 × 10^18^/n	0.72	2.55

## Data Availability

The original contributions presented in the study are included in the article, further inquiries can be directed to the corresponding author.
